# Robot-assisted submandibular gland excision via modified facelift incision

**DOI:** 10.1186/s40902-017-0122-4

**Published:** 2017-09-05

**Authors:** Seung Wook Jung, Young Kwan Kim, Yong Hoon Cha, Yoon Woo Koh, Woong Nam

**Affiliations:** 1 Department of Oral and Maxillofacial Surgery, Yonsei University, College of Dentistry, Seoul, Korea; 2 Department of Otorhinolaryngology, Yonsei University, College of Medicine, Seoul, Korea

**Keywords:** Robot-assisted surgery, da vinci Xi, Submandibular gland, Sialolithiasis, Modified facelift incision

## Abstract

**Background:**

The conventional transcervical resection for submandibular gland disease has some risks and an unsatisfactory cosmetic result. Recently, robot-assisted surgery has been developed as a plausible substitute for conventional surgery which provides an excellent cosmetic outcome.

**Case presentation:**

The authors performed robot-assisted sialadenectomy via modified facelift incision using the da Vinci Xi surgical system (Intuitive Surgical Inc., CA, USA) with two endowrist arms (monopolar curved scissors and Maryland bipolar forceps) successfully in a 44-year-old female patient who suffered from sialolith and severe atrophic submandibular gland.

**Conclusions:**

If similar studies are done in the future, this robot-assisted sialadenectomy may become established as an alternative to existing disadvantageous surgical methods.

## Background

The submandibular gland is vulnerable to non-neoplastic disorders (sialolithiasis and sialadenitis) due to its anatomic characteristics. The most common benign neoplasm is pleomorphic adenoma, and tumors of the submandibular gland are infrequently malignant [1]. The conventional treatment method of transcervical resection has some risks such as paresis of the marginal branch of the facial nerve, lingual nerve paresis, xerostomia, and an unsatisfactory cosmetic result [2]. Notwithstanding various techniques such as intraoral resection [3, 4] and endoscopic-assisted resection [5, 6] to reduce these complications, there are still postoperative discomforts, such as a temporary lack of function of lingual nerve and a temporary limitation of tongue movement [3]. Recently, robot-assisted surgery has been developed as a plausible substitute for conventional surgery which provides an excellent cosmetic outcome [7, 8]. Earlier robot-assisted surgeries were performed via a retroauricular approach [7], recent surgeries are being performed via modified facelift incision (MFI) approach [9–11], the postoperative scar being completely hidden by the auricle and hair. In this paper, the authors report a case of robot-assisted submandibular sialoadenectomy via MFI.

## Case presentation

**Fig. 1:**
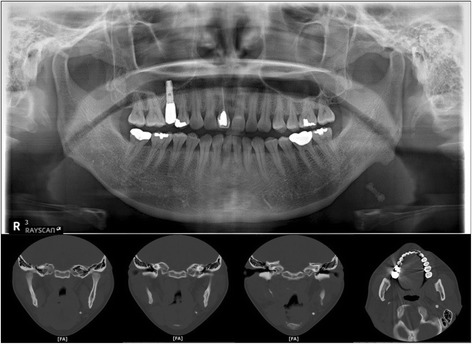
The panoramic radiograph showed an elongated radiopaque structure imposed on the *left* submandibular area (*top*). Computerized tomographic (CT) scan of the mandibular region showed the presence of multiple high attenuated materials and elongated sialolith located within the *left* Wharton’s duct. Very severe atrophic submandibular gland was also found (*bottom*).

A 44-year-old female presented with a chief complaint of 3-year history of recurrent pain and intermittent swelling to the left mandibular region. The swelling was usually worsened by meals, extreme pain arising once a month. When the pain started, it lasted about 10 min, with an NRS (numeric rating scale) score of 10. She had recently begun to have pain every 4 h. On examination, there was a tense and sensitive submandibular salivary gland and visible swelling in the posterior part of the left side of submandibular area. No salivary flow was appreciated from the left submandibular duct. The radiograph showed an elongated radiopaque structure imposed on the left submandibular area (Fig. 1 top). Computerized tomographic (CT) scan of the mandibular region showed the presence of multiple high attenuated materials and elongated sialolith located within the left Wharton’s duct. Also, very severe atrophic submandibular gland was found (Fig. 1 bottom).

**Fig. 2:**
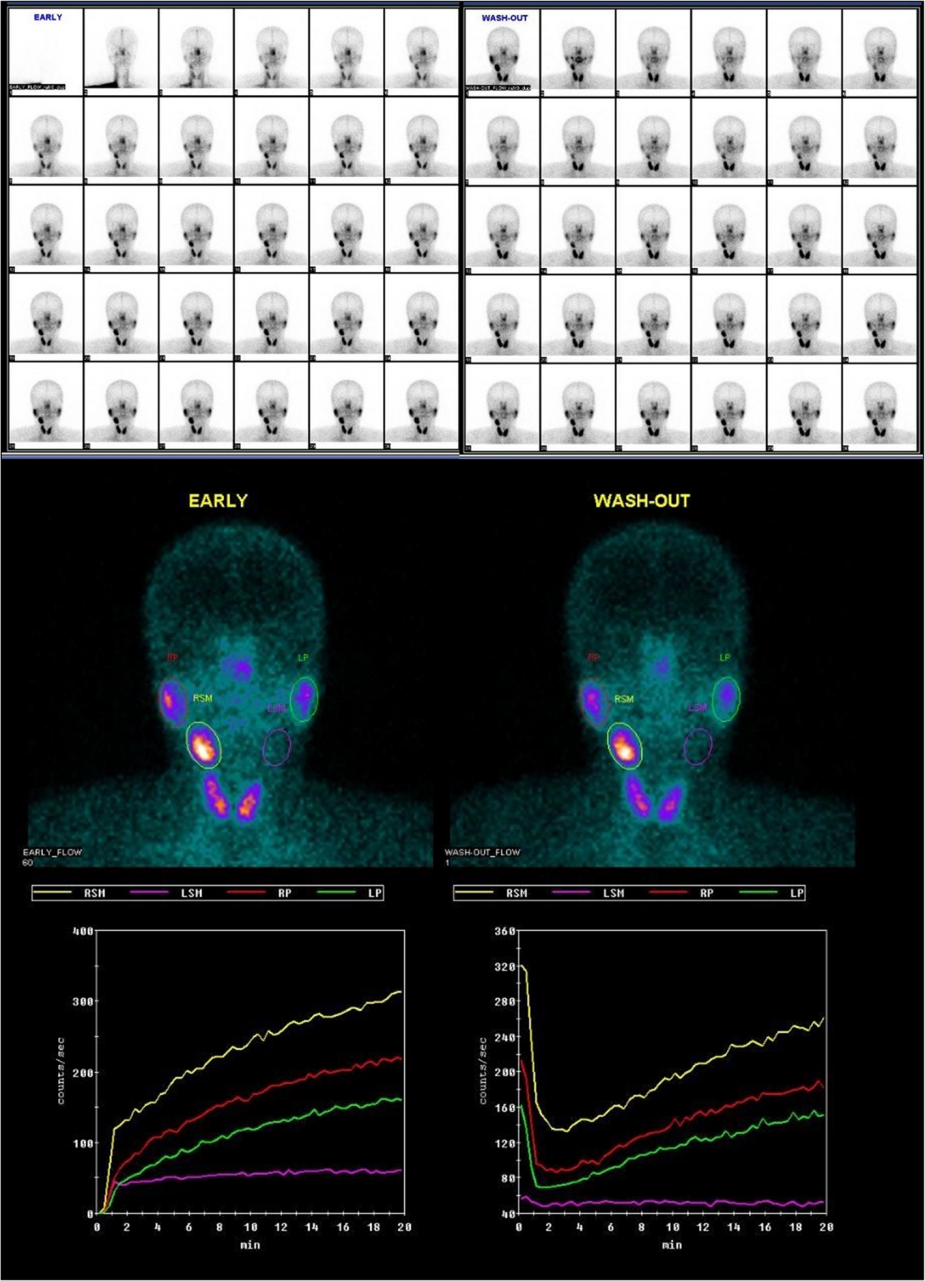
Preoperative technetium-99m pertechnetate salivary gland scintigraphy revealed that right salivary glands were within normal limits but with no definite radiotracer excretion in the Lt. submandibular gland

Preoperative technetium-99m pertechnetate salivary gland scintigraphy revealed that other salivary glands were within normal limits, but with no definite radiotracer excretion in the Lt. submandibular gland (Fig. 2).

### Surgical procedures

**Fig. 3:**
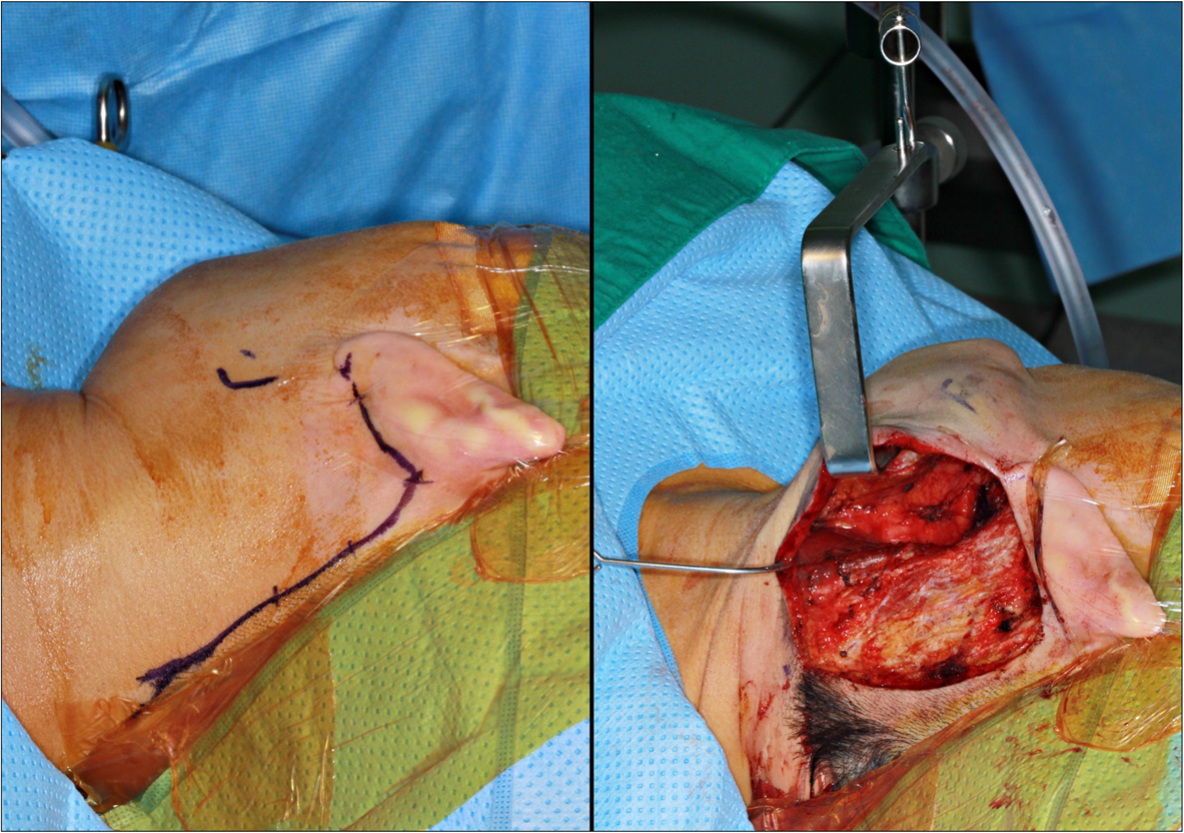
Modified facelift incision (MFI) for robot-assisted submandibular gland excision (*left*) and obtaining a sufficient amount of working space (approximately 10-cm height) for securing a self-retaining retractor (*right*)

The patient was placed on the operating table in supine position and was induced with short-acting paralytics to allow for monitoring of the branches of the facial nerve during dissection. General anesthesia was obtained via oral endotracheal intubation. The neck was extended with the placement of a shoulder roll, and the head was turned to the opposite side of the involved parotid. The patient was prepared and draped in a sterile fashion. The ipsilateral commissure of the mouth was prepared as readily visible. The incision line was marked (standard modified facelift incision). 2% lidocaine with epinephrine was injected within the subcutaneous tissues of the proposed surgical incision, involving a standard preauricular curvilinear incision which begins at the tragus, going around the inferior border of the lobule and then continuing backwards in the auriculomastoid groove. The superior aspect of the postauricular incision reached to the level of the superior aspect of the mastoid and then was extended posteriorly into the hair line of the neck for cosmesis (Fig. 3 left).

**Fig. 4:**
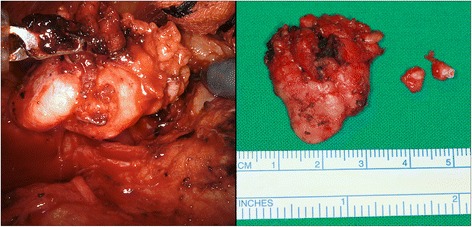
Dissection using the da Vinci Xi surgical system (Intuitive Surgical Inc., CA, USA) with two endowrist arms (monopolar curved scissors and Maryland bipolar forceps) (*left*) and excised specimen (*right*)

After skin incision, the subplatysmal skin flap is elevated just above the sternocleidomastoid (SCM) muscle using a monopolar electrocautery under direct vision. The greater auricular nerve and external jugular vein can be identified located superficial to the SCM muscle. The skin flap is elevated until the anterior extent reaches the midline of the anterior neck, the superior extent the inferior border of the mandible and the inferior extent the level of omohyoid muscle. Skin flap elevation below the mandible should be performed carefully to minimize injury to the nearby marginal branch of the facial nerve. Normally two assistant surgeons are required to comfortably lift up the skin flap with an Army-Navy retractor or a right-angle breast retractor. After obtaining a sufficient amount of working space (approximately 10-cm height), a self-retaining retractor is applied through the space and is secured [12, 13] (Fig. 3 right). Dissection began at the lower border of the SMG using the da Vinci Xi surgical system (Intuitive Surgical Inc., CA, USA) with two endowrist arms (monopolar curved scissors & Maryland bipolar forceps) (Fig. 4 left). The proximal facial artery was ligated with vascular clips, the lingual nerve was separated from the submandibular ganglion with monopolar cautery, and Wharton’s duct was ligated with a vascular clip. The lingual and hypoglossal nerves were well preserved. The specimen was well excised, the surgical bed irrigated with warm saline and bleeding control under both robot view and direct vision was performed (Fig. 4 right). A close suction drain was inserted posterior to the hairline incision, and the wound was closed with Dermabond skin adhesive (Ethicon, USA) after subcutaneous layer suture. The pathologic report was sialolith with ductal atrophy. There was no postoperative complication.

## Conclusions

**Fig. 5:**
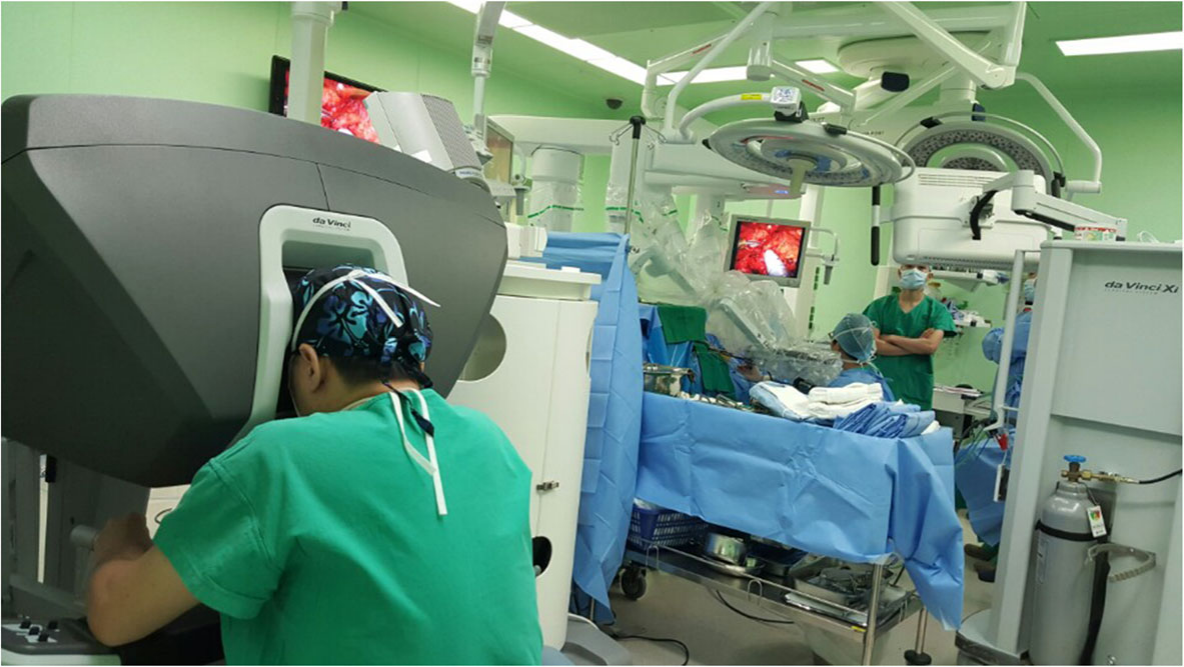
The da Vinci Xi surgeon console

**Fig. 6:**
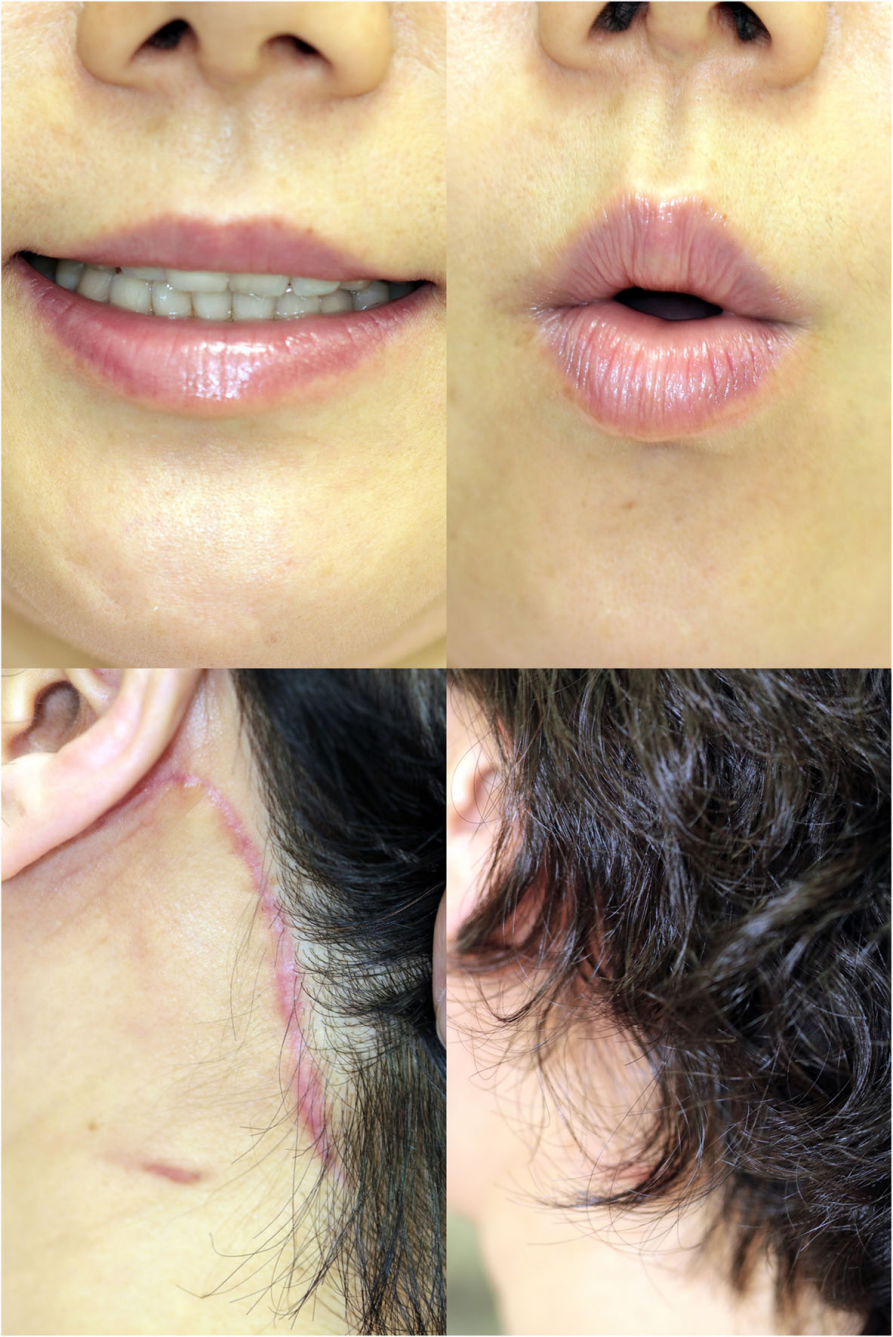
The patient shows stable nerve function and esthetic result at postoperative 3 months

Since Terris et al. reported that modified facelift incision (MFI) is an alternative approach to parotidectomy for selected patients [14], there have been many reports on the versatility and esthetic advantages of MFI in various surgeries [15–21]. Various approaches have been proposed for the application of robotic surgery to the neck [22–24]. Since robotic cervical surgery using MFI was reported by Koh et al. [10], the usefulness of this approach has been affirmed, and even the cervical lymphadenectomy, is now being performed using a robot [25–28]. In this case, enough space was secured for robot operation during the approach using MFI, leaving a scar which was largely concealed by hair. Because the authors have already published a paper on endoscopic cervical lymphadenectomy [29], the advantages and disadvantages of using robots and endoscopes are clear to them. Compared with endoscopes, robots are more flexible, allowing for more free tissue detachment and the ability to perform uncomplicated operations with two arms. Three arms make operations much easier. In addition, it is possible to perform surgery in a more comfortable sitting position on the surgical console (Fig. 5) and since the visual field is three-dimensionally detailed and bright, it is possible to observe microscopic nerves and blood vessels rather than view them directly transcervically. Several types of robotic arms have been developed, but this operation is possible with only two types—monopolar curved scissors and Maryland bipolar forceps, ligation of blood vessels made possible with a robot arm or vascular clip. However, it cannot be felt when a structure like a mandible that restricts the movement of a robot arm is touched, so it is considered as a disadvantage that a surgical assistant should always observe it from the side. The cost is not likely to be an obstacle in choosing surgery, as patients have recently had a range of private insurance. The operation time was 3 h and 11 min, and it was not worse than open surgery for 2 h except for suture time. If one is familiar with endoscopic surgery, there should be no great difficulty. No specific postoperative complications were reported. In this case, the patient was discharged after the hemo-Vac discharge was reduced to 20 ml/day without any postoperative complications and showed great satisfaction with the operation results (Fig. 6). If similar studies are done in the future, this method may become established as an alternative to existing disadvantageous surgical methods.
